# From Metabolic Syndrome to Neurological Diseases: Role of Autophagy

**DOI:** 10.3389/fcell.2021.651021

**Published:** 2021-03-19

**Authors:** Jessica Maiuolo, Micaela Gliozzi, Vincenzo Musolino, Cristina Carresi, Federica Scarano, Saverio Nucera, Miriam Scicchitano, Francesca Bosco, Stefano Ruga, Maria Caterina Zito, Roberta Macri, Rosamaria Bulotta, Carolina Muscoli, Vincenzo Mollace

**Affiliations:** ^1^IRC-FSH Department of Health Sciences, University “Magna Graecia” of Catanzaro, Catanzaro, Italy; ^2^IRCCS San Raffaele, Rome, Italy

**Keywords:** metabolic syndrome, vascular endothelium, neurological disorders, autophagy, brain-derived neurotrophic factor

## Abstract

Metabolic syndrome is not a single pathology, but a constellation of cardiovascular disease risk factors including: central and abdominal obesity, systemic hypertension, insulin resistance (or type 2 diabetes mellitus), and atherogenic dyslipidemia. The global incidence of Metabolic syndrome is estimated to be about one quarter of the world population; for this reason, it would be desirable to better understand the underlying mechanisms involved in order to develop treatments that can reduce or eliminate the damage caused. The effects of Metabolic syndrome are multiple and wide ranging; some of which have an impact on the central nervous system and cause neurological and neurodegenerative diseases. Autophagy is a catabolic intracellular process, essential for the recycling of cytoplasmic materials and for the degradation of damaged cellular organelle. Therefore, autophagy is primarily a cytoprotective mechanism; even if excessive cellular degradation can be detrimental. To date, it is known that systemic autophagic insufficiency is able to cause metabolic balance deterioration and facilitate the onset of metabolic syndrome. This review aims to highlight the current state of knowledge regarding the connection between metabolic syndrome and the onset of several neurological diseases related to it. Furthermore, since autophagy has been found to be of particular importance in metabolic disorders, the probable involvement of this degradative process is assumed to be responsible for the attenuation of neurological disorders resulting from metabolic syndrome.

## Introduction

Metabolic syndrome (MetS), also known as syndrome X, insulin resistance syndrome, or Reaven syndrome; is not a single pathology, but a constellation of cardiovascular disease risk factors. These clinical conditions comprise: (a) central and abdominal obesity, (b) systemic hypertension, (c) insulin resistance (or type 2 diabetes mellitus), and (d) atherogenic dyslipidemia, the so-defined “deadly quartet” (McCracken et al., 2018). There are several definitions to indicate the characteristics of MetS; among these, we point out the one provided by the International Federation of Diabetes (IDF) of 2006 (Saklayen, 2018), which states that metabolic syndrome is represented by glucose in the blood above 5.6 mmol/L (100 mg/dl) (or diabetes already diagnosed) along with the presence of two or more of the following conditions:

• HDL cholesterol (HDL-C) < 1.0 mmol/L (40 mg/dl) in men; < 1.3 mmol/L (50 mg/dl) in women or drug treatment for low HDL-C;
• blood triglycerides > 1.7 mmol/L (150 mg/dl) or drug treatment for elevated triglycerides;
• blood pressure > 130/85 mmHg or drug treatment for hypertension;
• waist > 94 cm (men) or > 80 cm (women). Obesity is diagnosed using waist circumference, which correlates better with visceral adiposity than Body Mass Index (BMI) (Alberti et al., 2005).

**FIGURE 1:**
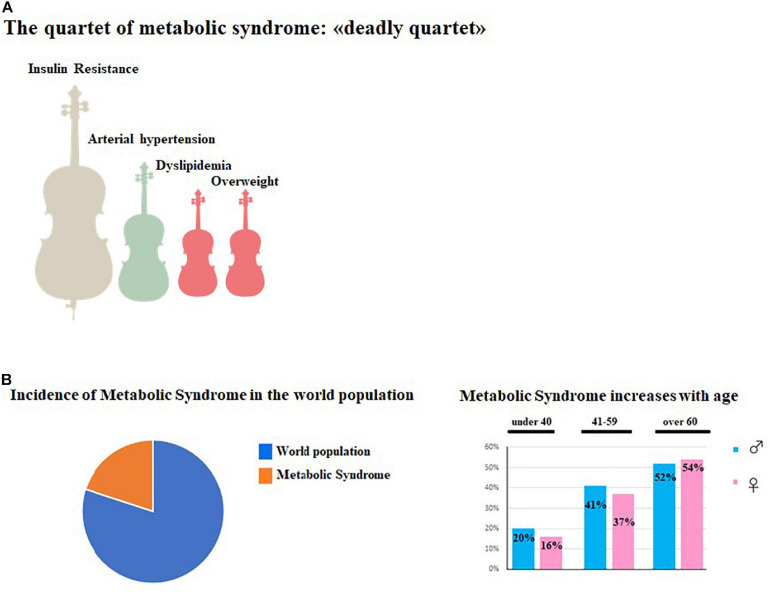
Main features of MetS. **(A)** represented the symptomatological quartet most widespread in MetS consisting of Insulin Resistance, Hypertension, Dyslipimedia and Obesity. **(B)** (Left) shows the global prevalence of MetS in the world population, while in **(B)** (right) the increase of MetS in aging men and women is shown.

The presence of three of the five main risk factors is sufficient to confirm a MetS diagnosis (Altieri et al., 2015). All other MetS definitions are very similar; however, parameters indicated may vary by a few units. The global incidence of MetS is very high, afflicting one third of adults 18 years or older in the United States alone (Aguilar et al., 2015). To date, we can estimate the global prevalence to be about one quarter of the world population (Li et al., 2008). In general, the prevalence of MetS has been found to increase with age, involving about 20% of males and 16% of females under 40 years of age; 41% of males and 37% of females between the ages of 40–59; and 52% of males and 54% of females over 60 years of age (Ervin, 2009; Aoki et al., 2014). During the last 15 years, the prevalence of MetS has increased (Pucci et al., 2017) and the main motivation is related to significant changes in lifestyle; the western lifestyle consists of numerous risk factors such as: high-fat diet, cigarette smoking, alcohol consumption, obesity, and physical inactivity (O’Doherty et al., 2016; Sarmiento Quintero et al., 2016; Finicelli et al., 2019). In particular, the western diet is based on high consumption of salt, refined sugars, and saturated fats that determine significant effects on body composition and metabolism such as: increased BMI, generalized and abdominal obesity, dyslipidemia, and type 2 diabetes (Misra and Khurana, 2008). In addition to lifestyle changes, we should include other concomitant factors such as: chronic inflammation, endothelial dysfunction, genetic susceptibility, hypercoagulability, and chronic stress (Srikanthan et al., 2016; Motamedi et al., 2017; Woo et al., 2019). The main features of MetS are summarized in Figure 1.

The first part of this review, after describing the main features of MetS, deepens the involvement of the vascular endothelium in this metabolic disorder based on current scientific literature. Subsequently, the development of neurological disorders resulting from metabolic syndrome is considered. Lastly, the importance of autophagic function has discussed with the aim of proposing this element as molecular target to reduce and/or eliminate the dangerous symptoms of MetS and the consequent neurological symptoms.

## The Role of the Endothelium in Metabolic Syndrome

MetS can, as already stated, increase the risk of developing type 2 diabetes mellitus, obesity, and cardiovascular diseases; contributing, therefore, to high rates of mortality and morbidity (Alberti et al., 2009). The common denominators between MetS and its metabolic disorders are chronic low-grade inflammation and activation of the immune system (Chawla et al., 2011; Ouchi et al., 2011; El-Benna et al., 2016). MetS demonstrates a central role in promoting tissue inflammation in the adipose tissue of the liver, muscles, and pancreas; with concomitant infiltration of macrophages, and production of pro-inflammatory cytokines including Tumor Necrosis Factor alpha (TNFα), Interleukin 6 (IL-6), IL1β, activation of the c-JUN N-terminal kinase (JNK), and nuclear factor-kappa B (NF-κB) pathways (Chawla et al., 2011; Elmarakby and Sullivan, 2012; Grandl and Wolfrum, 2018). Macrophages are classically classified into two distinct subtypes: the activated phenotype secreting pro-inflammatory cytokines (M1), and the alternatively activated phenotype which produces anti-inflammatory cytokines such as IL-10 (M2) (Chawla et al., 2011). Under normal conditions, macrophages have been described as being a mix between M1 and M2 phenotypes, while in MetS, a phenotypic shift from M2 to M1 has been noted, both in mice and in humans (Wentworth et al., 2010) which shows a marked proinflammatory response. The inflammatory process includes increased vascular permeability to immune cells with the endothelium playing a fundamental role in this process (Bañuls et al., 2017; Bruno et al., 2017; Friesen and Cowan, 2019). The endothelium is composed of a layer of cells and constitutes the internal lining of the blood vessels; playing the role of a selectively permeable barrier. Endothelial cells do not play a passive role; instead, they regulate very important physiological functions such as: maintaining homeostatic balance, controlling vasomotor tone, ensuring proper permeability, and managing innate immunity reactions (Godo and Shimokawa, 2017). Precisely for this reason, the endothelium can be considered as an organ. The permeability of the endothelium allows the transport of only a few necessary molecules; this selectivity is ensured by a fine regulation carried out by junction proteins that have the function of keeping endothelial cells closely adjacent; thereby preventing the passage of unwanted molecules or cells (Babushkina et al., 2015). Junction proteins can be classified into two categories: tight junctions, also known as zonula occludens, and adherens junctions. Ensuring the cell adhesions and junctions, cytoskeleton proteins play a particularly key role (Maiuolo et al., 2018). When the expression of these proteins is nullified or reduced, endothelial disassembly and dysfunction occurs (Kiseleva et al., 2018).

Vascular endothelium cells (VEC) play a fundamental role in the human body; ensuring the regulation of vasomotor functions, the maintenance of vessel walls, anti-platelet aggregation, and endocrine functions. Numerous harmful stimuli can lead to endothelial cell dysfunction (ECD) with consequential increases in the risk of many diseases including cardiovascular diseases (Vykoukal and Davies, 2011; Khaddaj Mallat et al., 2017; Kaplan et al., 2018; Maiuolo et al., 2020a). In MetS patients, the ECD is a specific early pathophysiological indicator of cardiovascular disorders; its recognition and early intervention are of extreme importance in the prevention, treatment, and prognosis of cardiovascular diseases (Wei et al., 2014; Dalal et al., 2020). In particular, many substances secreted by VEC are considered important indicators of the function of endothelial cells, these include: Plasminogen Activator Inhibitor-1 (PAI-1), von Willebrand factor (vWF), vascular endothelial cadherin (VE-cad), Thrombomodulin (TM), and Vascular endothelial growth factor (VEGF). PAI-1 is mainly produced by the endothelium but is also secreted by other types of tissue, such as adipose tissue. PAI-1 is a fibrinolysis inhibitor; preventing the physiological process that degrades blood clots and contributing to the formation of atherosclerotic plaques (Deng et al., 2012; Yarmolinsky et al., 2016). vWF is a macromolecular plasma glycoprotein involved in hemostasis with important implications in blood viscosity; an increase can predict the risk of thrombosis (Shahidi, 2017). VE-cad is a classic cadherin, belonging to the cadherin superfamily; it is expressed specifically on the endothelial surface and concentrated in existing cell-cell junctions. VE-cad is known to be necessary to maintain a restrictive endothelial barrier; early studies, using blocking antibodies against VE-caderin, resulted in increased endothelial permeability *in vitro* (Hakanpaa et al., 2018) and in hemorrhage *in vivo* (Corada et al., 2002). TM is an integral membrane protein expressed on the surface of endothelial cells and serves as a cofactor for thrombin. TM reduces blood clotting by converting thrombin from a pro-clotting factor to an anti-coagulant factor (Loghmani and Conway, 2018). In normal conditions, plasma TM levels are low; but when vascular endothelial cells are damaged, TM increases significantly; highlighting that its level can be used as a marker of endothelial injury (Stadtmann et al., 2011; Kishi et al., 2020). VEGF is a selective signaling protein that promotes the growth of new blood vessels and restores the supply of oxygenated blood to cells and tissues that have been deprived due to impaired blood circulation. VEGF also improves vascular permeability by increasing exudation of blood components and inflammatory cytokines (Sack et al., 2016). Scientific studies have shown that serum levels of PAI-1, vWF, VE-cad, TM, and VEGF were increased in patients with MetS compared to healthy individuals (Georgieva et al., 2004; Hajiluian et al., 2017; Mazidi et al., 2017; Wang et al., 2018). Moreover, an increase has also been found in children and adolescents with MetS, evidencing that these parameters can be considered predictors of early vascular changes (Wei et al., 2014). MetS negatively affects the function of the vascular endothelium; increasing vasoconstriction and prothrombotic state through different mechanisms (Aggoun, 2007; O’Shea et al., 2015; Pomero et al., 2015; Domingueti et al., 2016):

• Hyperglycemia, hyperlipemia, and hypertension increase the release of numerous cytokines, such as: IL-1β, IL-6, and growth factor PDGF; triggering the process of endothelial dysfunction (Varghese et al., 2018).
• Carbohydrate metabolism (hyperinsulinemia/insulin resistance or hyperglycemia/diabetes), dyslipidemia, obesity, and hypertension can all determine vascular contractility (Cuspidi et al., 2018).
• Altered carbohydrate metabolism, dyslipidemia, hypertension, and obesity lead to increased PAI-1, vWF, VE-cad, TM, and VEGF production; a condition responsible for impaired anticoagulant and fibrinolytic activity which can cause hypercoagulability: a low fibrinolytic and high viscosity state, and promote the formation of microthrombi (Wei et al., 2014).

**FIGURE 2:**
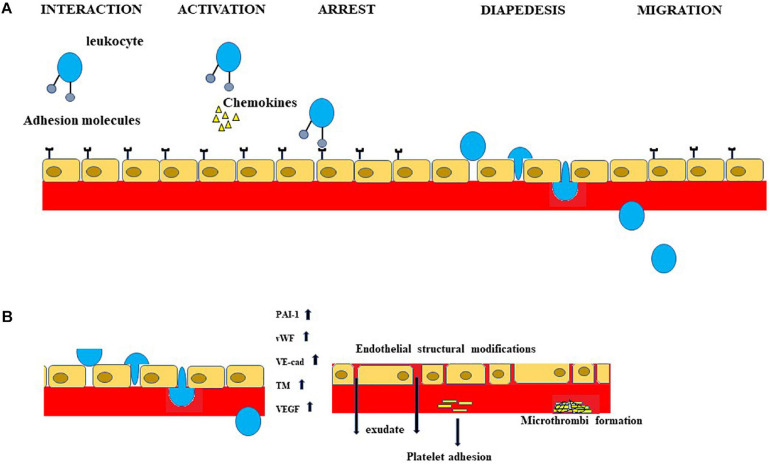
Inflammation of the endothelium is involved in MetS and subsequent neurological manifestations. A portion of the inflammatory process, occurring both in MetS and in neurological disorders related to MetS, is schematized in Figure. In particular, **(A)** shows the sequence of leukocyte extravasation across the endothelium. **(B)** Important indicators of the dysfunction of endothelial cells are shown. The increases in PAI-1, vWF, VE-cad, TM and VEGF determine endothelial structural modifications, exudate production, platelet adhesion, and microthrombi formation.

The main stages of endothelial inflammation, involved in Mets are represented in Figure 2. In the light of what has been stated, a clinical evaluation of endothelial involvement should be carried out both in patients with MetS and in people not affected who show clear risk factors related to body weight, metabolism of carbohydrates, and blood pressure. In this way, it could be possible to prevent and control secondary disorders of MetS.

## From Metabolic Syndrome to Neurological Diseases

The effects of MetS are multiple and wide ranging; some of which have an impact on the central nervous system (CNS); causing neurodegenerative and neurological diseases (Kathy et al., 2012; Błaszczyk and Gawlik, 2016; Van Dyken and Lacoste, 2018). The CNS is the most complex and organized system in the human body and proper neuron functioning requires a correct biochemical balance (which includes suitable chemical and electrical signaling) in order to support and maintain adequate intracellular and intercellular communication. In addition, the correct concentration of ionic and signaling molecules must be guaranteed to remove catabolites and maintain a low concentration of neurotoxic mediators (Corasaniti et al., 2007; Maiuolo et al., 2018). A key role is played by the Blood Brain Barrier (BBB), which provides all these functions and represents a physical, selective, and highly lipophilic barrier; protecting brain tissue and separating it from systemic circulation (Daneman and Prat, 2015). The basic structure of the BBB consists of endothelial cells, tightly joined by junction proteins; while other constituents, including the basal membrane (BM), pericytes, and astrocytes, perform support and regulation functions (Andreone et al., 2015; O’Brown et al., 2018). The BBB endothelium is rich in specific proteins that act as carriers and receptors; responsible for the passage of metabolites, macronutrients, micronutrients, and junction proteins; which significantly limit their intercellular exchange. Endothelial cells consist of five or six times more mitochondria than other tissues in the human body as these organelles provide the energy required for endothelial cells to maintain cerebral homeostasis (Gentil et al., 2005). Endothelial dysfunction leads to “frailty” of the BBB; characterized by increased vascular permeability, impaired ability to preserve brain tissue homeostasis, infiltration of toxic blood-derived molecules, cells, microbial agents; which together trigger inflammatory and immune neurodegeneration (Maiuolo et al., 2018, 2019). Basement membrane (BM) is composed of collagen, laminin, heparin, and other glycoproteins; it determines an additional barrier in the BBB, although it can be interrupted by metalloproteinases of the matrix (Thomsen et al., 2017). Pericytes are in contact with the BM, juxtaposed to the endothelial cells, with which they are connected and are involved in the regulation of angiogenesis, vascular stability, and BBB control (ElAli et al., 2014). Astrocytes regulate vasomotor responses and the brain blood flow as a result of changes in neural activity; they also release regulating factors for the maturation and maintenance of the BBB (Lacoste and Gu, 2015). Integrity of the BBB is essential to ensure the proper functioning of the CNS and many neurological disorders are caused by its rupture (Zhao et al., 2015; Chakraborty et al., 2017; Maiuolo et al., 2020b). The fundamental cause of the loss of barrier integrity is the inflammatory process that occurs in the CNS as a result of neuronal damage (Varatharaj and Galea, 2017). Under normal conditions, the integrity of the BBB prevents the passage of immune cells into the CNS; however, inflammation induces the opening of the BBB thereby altering the various components. The main consequences are:

• release of cytokines and inflammation mediators (Incalza et al., 2018; Oppedisano et al., 2020);
• leukocyte extravasation (diapedesis) across the endothelium (Rudziak et al., 2019);
• destruction of BBB cells (Chen et al., 2019).

The first important step for the diapedesis is the interaction between the leukocytes and adhesion molecules on endothelial cells, including P-selectin (a cell adhesion molecule on the surfaces of activated endothelial cells); E-selectin (endothelial-leukocyte adhesion molecule 1 expressed only on endothelial cells and activated by cytokines) (Laird et al., 2018); ICAM-1 (intercellular adhesion molecule-1 typically expressed on endothelial cells and cells of the immune system) (Schaefer et al., 2017); and VCAM-1 (vascular cell adhesion molecule-1 that mediates the adhesion of lymphocytes, monocytes, eosinophils, and basophils to vascular endothelium) (Fan et al., 2019). The next step involves the rolling of leukocytes along the wall of the vessel; the release of chemokines that strengthen contact with the endothelium; and the extension of pseudopods, from leukocytes, that allow the attack of endothelial cells (Lutz et al., 2017). The leukocyte crossing of the endothelium is defined as “transendothelial cell migration” and the correct direction to follow is provided by chemotactic and haptotactic stimuli outside of the vascular lumen and beyond. This movement occurs at the same time as the morphological variations of the leukocytes, which guide the cell nucleus through tight endothelial junctions and pores. Transendothelial leukocyte migration can take place through junctions between adjacent endothelial cells (paracellular migration) or through the body of the endothelium (transcellular migration) (Nourshargh and Alon, 2014). Although paracellular migration occurs in 70–80% of cases, the cerebral vascular endothelial cells appear to be an exception to this rule and prefer transcellular migration. This phenomenon is motivated by the particularly narrow specialized junction structures expressed by the brain endothelial cells (Engelhardt and Ransohoff, 2012). It has been widely demonstrated that increased inflammation facilitates BBB breakage (Varatharaj and Galea, 2017); with several mechanisms that include: downregulation of many junction proteins and important amino acid transporters (Yoo et al., 2016), alteration of transcytosis (Ransohoff et al., 2015), up-regulation of transporters for TNF-α, lysosomal degradation enzymes, VCAM-1 P/E-selectin (Varatharaj and Galea, 2017), and the accumulation of insoluble fibrin; responsible for the alteration of the immune response and blood clotting (Davalos and Akassoglou, 2012). Ultimately, these activated inflammatory processes are responsible for the destruction of cellular components of the BBB. In particular, the integrity of astrocytes is compromised, the leukocyte infiltration is increased, and the input of pathogens and toxins in the central nervous system is allowed (Van Dyken and Lacoste, 2018). Astrocytes are very susceptible to oxidative stress and inflammation; becoming unable to perform their role of maintaining ions and neurotransmitters in physiological conditions for BBB integrity (Bhat et al., 2012).

A positive feedback cycle has recently been described where activated microglia produce ROS; leading to cell death and increased levels of local glutamate. This condition results in increased secretion of proinfiammatory cytokines, a further activation of microglia, and destruction of endothelial BBB cells (Mittal et al., 2014; Cai et al., 2017). Increased systemic inflammation impacts many of the systems in the body including the brain, in fact, inflammation is closely associated with neuropathology (Fan et al., 2014). To date, a series of epidemiological studies have shown that MetS increases the risk of developing neurodegenerative diseases, CNS dysfunction (Islam, 2017; Motamedi et al., 2017; Ricci et al., 2017; Arshad et al., 2018; Karaca and Karaca, 2018; Palta et al., 2021), and reduced cognitive performance including deficits in memory, visuospatial abilities, executive functioning, processing speed, and overall intellectual functioning. MetS was found to be a factor of risk for: ischemic stroke, intracranial arteriosclerosis, periventricular white matter hyperintensities, and subcortical white matter lesions (Yates et al., 2012). In this regard, changes in brain metabolism have been shown to be responsible for the onset of neuroinflammation; these brain changes may represent an early associated brain impairment with peripheral metabolic disorders.

### MetS: Obesity, Diabetes, and Cognitive Functions

Obesity is the excess accumulation of body fat caused by an imbalance between energy intake and consumption. The effects of obesity are largely mediated through inflammation and, in these mouse models, neural inflammation can be detected even earlier than weight gain (Van Dyken and Lacoste, 2018). Obesity is directly related to impairment of cognitive function and an increased risk of different forms of dementia. Clinical and experimental evidence indicates that obesity and/or a high-fat diet is associated with impairment of learning, memory, and executive functioning (Anstey et al., 2011; Bremer and Jialal, 2013; Miller and Spencer, 2014; Saltiel and Olefsky, 2017; Costello and Petzold, 2020). Many studies have been carried out on the correlation of BMI-cognitive function and waist circumference-cognitive function. In general, it has been found that BMI is inversely related to cognitive function, including memory and executive functioning. In addition to cognitive performance, obesity can affect brain structure; leading to atrophy (Climie et al., 2015; Gogniat et al., 2018). Moreover, a relationship has also been described regarding particular areas of the brain; the temporal and frontal lobes appear to be particularly vulnerable to the effects of obesity and gray matter volumes of these brain regions are reduced in obese patients; resulting in the reduction of neuronal viability (Gómez-Apo et al., 2018; Lee et al., 2020). The first area of the brain to be affected is the hypothalamus; the subsequent damage reduces the number of synapses on hypothalamic neurons and increases neural apoptosis (Sohn, 2015). The obese mice showed a lower yield than the control group, thus confirming involvement of the hippocampus and cognitive impairment (Pendlebury and Rothwell, 2009; Hargrave et al., 2016; Van Dyken and Lacoste, 2018).

The activation of the inflammatory process, present in obesity, seems to be the fundamental cause of the alteration of the health of the brain (function and structure). In particular, a chain reaction occurs in which the activation of transcription factor NF-kB can be appreciated (Jais and Brüning, 2017), followed by upregulation of pro-inflammatory cytokines, such as: IL-1β, TNF-α, and IL-6 (Lawrence, 2009). It is also important to note that the observed cognitive decline was preceded by the reduction of protein TJ expression and loss of BBB integrity (Gustafson et al., 2007; Davidson et al., 2013; Dorfman and Thaler, 2015).

Scientific evidence has shown, both in animal models and in humans, a close correlation between diabetes mellitus [type 1 (T1DM) and type 2 (T2DM)] and cognitive decline leading to dementia; although T2DM has shown a stronger association with brain disorders (Monette et al., 2014; Zilliox et al., 2016; Black et al., 2018). Among the components of MetS, hyperglycemia has the strongest association with the risk of developing cognitive deterioration (Šmahelová, 2017). Numerous studies have shown reduced performance in cognitive activities in diabetic compared to non-diabetic controls which include memory, mental speed, mental flexibility, and executive function. These dysfunctions have been correlated with a reduced density of gray matter of the prefrontal and temporal cortex (van den Berg et al., 2010). It is not perfectly clear when cognitive impairment occurs in the course of diabetes; in some cases these events can be very early, while in other cases they are later events (Hassing et al., 2004). It is known that insulin signaling improves synaptic plasticity in the hippocampus; playing an important role in memory and learning. In fact, insulin facilitates long-term enhancement of the hippocampus (LTP) and, in the healthy mammalian brain, is associated with learning and memory; increasing the expression of N-methyl-D-aspartate receptors. In addition, insulin regulates the concentration of several important neurotransmitters in memory maintenance such as: acetylcholine, norepinephrine, and epinephrine (Boyd et al., 1985; Figlewicz et al., 1993; Patterson et al., 2016). Insulin dysregulation in patients with diabetes could facilitate cognitive disorders (Kullmann et al., 2016; Denver et al., 2018; Tumminia et al., 2018). Insulin in the brain also has the function of regulating mitochondria (Heras-Sandoval et al., 2012). It has been shown that, in a condition of overt diabetes, highly altered insulin acts on the pre-synaptic terminals causing the mitochondrial DNA mutations responsible for functional and structural changes of the organelles (Lu et al., 2004). Dysfunction of the mitochondria causes the depletion of energy reserves; the enzymatic complexes of the electronic transport chain (complexes I and III) are altered, leading to neuronal synaptic loss and cognitive deficits (Kim et al., 2009; Choi et al., 2014).

### MetS: Cognitive Dysfunction and the Possible Role of Brain-Derived Neurotrophic Factor

In the early 1950s, the neurotrophic theory was developed. This theory is based on the functional mechanisms adopted by effecter cells to control growth, survival, differentiation, and neural function through the production of biomolecules, identified as neurotrophins (Lewin and Barde, 1996; Dechant and Neumann, 2002). To date, it is known that the family of these molecules includes: Nerve Growth Factor (NGF), Brain-Derived Neurotrophic Factor (BDNF), Neurotrophin3 (NT3), NT4, NT5, NT6, and NT7 (Emanueli et al., 2014; West et al., 2014; Skup, 2018). BDNF is the most abundant neurotrophin in the mammalian CNS and is synthesized and expressed in different cerebral regions. BDNF is a protein encoded by the *BDNF* gene found in humans on chromosome 11; it is initially synthesized as a precursor, pro-BDNF, consisting of 129 amino acids in the endoplasmic reticulum. Subsequently, pro-BDNF is split through the action of proconvertase; in a mature form of 118 amino acids in the trans-Golgi. The mature shape dimerizes; forming the BDNF active factor (Hempstead, 2015). BDNF is synthesized in the cytoplasm of neurons and glia; although it is also found in the skeletal and smooth muscles, liver, lymphocytes, endocrine system, pancreas, endothelial cells, and adipose tissue (Ansari et al., 2016). BDNF is involved in many neurological processes such as: neural growth, differentiation, synaptic conductivity, plasticity, neurogenesis, neuroregeneration, cognition, memory, learning, and dendrite growth (Smith et al., 2015; Morales-Marín et al., 2016). For this reason, structural and functional alteration of BDNF, determined by several factors, has been implicated in a number of neurodegenerative diseases and psychiatric disorders such as Alzheimer’s disease, Huntington’s disease, Parkinson’s disease, schizophrenia, intellectual disability, autism, depression, and the development of mood disorders (Björkholm and Monteggia, 2016). It has been shown that neurotrophins exert a metabotropic effect with respect to glucose, lipid, and energy; therefore, they have an important role in MetS (Chaldakov et al., 2014; Kim et al., 2020). Recent studies have shown a correlation between BDNF and MetS. In fact, there is a reduction of BDNF levels, especially in the stages of advancement of MetS (Morales-Marín et al., 2016). BDNF, which is reduced in MetS, performs critical functions within the CNS and alterations determine neurological diseases. Therefore, we can indirectly conclude that cognitive dysfunctions in MetS are correlated with BDNF. However, in order to better understand this topic, further studies should be carried out.

## Can Autophagy Interfere with Mets and Associated Neuronal Disorders?

Autophagy is a catabolic intracellular process evolutionarily preserved and finely regulated; essential for the recycling of cytoplasmic materials (proteins, lipids, carbohydrates) and for the degradation of damaged organelles (mitochondria, endoplasmic reticulum and peroxisomes), whose accumulation can be toxic to cells (Lapaquette et al., 2015). In this way, the renewal and proper functioning of intracellular organelles is also guaranteed. In lysosomes, autophagy determines: the recycling of cell portions that can still be used, the reduction of cell waste, the protection and maintenance of cellular energy and cellular adaptation to environmental challenges; all processes that contribute to cell survival (Madrigal-Matute and Cuervo, 2016). Therefore, autophagy is primarily a cytoprotective mechanism; even if excessive self-degradation can be detrimental. To date, it is known that autophagic activity is also connected to other important roles such as: the maintenance of cellular metabolism, control of cell cycle, immune response, development, and differentiation or cell death (Hu et al., 2019). In addition, when cells are subjected to a wide range of stressful conditions (physical, chemical, or metabolic), the autophagic process is activated in order to maintain cellular homeostasis. Since autophagy is critical for the maintenance of cellular metabolic homeostasis of whole body, its dysregulation may be the cause of the onset of pathologies which can affect liver, heart, brain, myopathies, diabetes, obesity, and cancer (Lim et al., 2018). Four main forms of autophagy have been described in mammalians:

• Macroautophagy (herein autophagy) is a catabolic process in which old cytoplasmic proteins, lipids, or damaged organelles are incorporated in double-membrane vesicular structures called autophagosomes. Autophagosomal membranes may derive from a number of sources such as: endoplasmic reticulum, Golgi apparatus, mitochondria, endosomes, and the plasma membrane. The movement of the autophagosomes takes place along the microtubules until reaching the lysosomes where the degradation process occurs (Feng et al., 2015).
• Microautophagy, a non-selective lysosomal process, refers to the direct engulfment of small amounts of cytosolic material (Li and Hochstrasser, 2020).
• Chaperone-mediated autophagy refers to the process in which some protein complexes are recognized by the cytosolic chaperones that deliver them to the surface of the lysosomes (Tekirdag and Cuervo, 2018).
• Selective autophagy that requires the respect of three criteria to ensure an efficient process (Zaffagnini and Martens, 2016): (i) the specific recognition of the cargo to be phagocytized; (ii) an efficient bonding of the cargo to a nascent autophagosome; (iii) the exclusion of components not part of the cargo.

The autophagy mechanism requires the expression of a set of evolutionarily conserved autophagy related genes (*ATGs*) whose protein products combine to form several useful complexes in the various stages of this process. All *ATG* genes, originally discovered in yeast, are needed for the efficient formation of autophagosomes that blend with the lysosomes, orchestrating and mediating the intra-cytoplasmic cargo degradation (Mizushima, 2018). The whole autophagic process is divided into six main steps which need to be tightly regulated both spatially and temporally: initiation, nucleation of vesicles, membrane elongation, closure, maturation, and degradation (Grumati and Dikic, 2018). In particular, the induction of autophagy occurs with the recruitment of Atg proteins and other proteic complexes in a specific subcellular position, called Phagophore Assembly Site, and with the nucleation of an insulating membrane forming a structure called phagophore (Yu et al., 2018). The very first autophagy-specific complex is termined Unc-51-Like Kinase 1 (ULK1) and is composed by ULK1 itself, Atg13, Focal adhesion kinase family Interacting Protein (FIP200) and Atg101 (Zachari and Ganley, 2017). When activated, ULK1 phosphorylates other autophagy pathway components, including Beclin1 and Atg9 and the onset of phagophore formation occurs when ULK1 complex is moved to a specific place in the endoplasmic reticulum membrane marked by the activated Atg9 protein (Levine and Kroemer, 2019). Elongation of the autophagosome membrane determines the expansion of the autophagosome in a sphere, around to the portion of the cytosol that must be degraded (Yoshii and Mizushima, 2017). This step involves Atg7 and Atg10 complexes that combine Atg12 and Atg5 proteins. The Atg12-Atg5 conjugate, along with Atg16L1, adds phosphatidylethanolamine monomers, determining the elongation of the autophagosome (Kauffman et al., 2018). The protein Atg9 is partly regulated by the Atg1 complex and other Atg proteins such as Atg17, Atg2, Atg18, Atg14L, Atg8, Atg21, Atg16, Atg12, and Atg5 (Nishimura and Tooze, 2020). The next step is the clearance of most Atgs proteins and the fusion of the autophagosome with the lysosomal membrane to form an autolysosome. Finally, the authophagic load is degraded by the hydrolytic environment into the autolysosome (Yu et al., 2018).

To date it is known that a dysfunction of autophagy is related to the onset of some diseases including cancer (White et al., 2015; Li Y. J. et al., 2017; Li et al., 2020), aging (Wong et al., 2020), neurodegenerative diseases (Menzies et al., 2015; Boland et al., 2018; Kumar et al., 2018; Suresh et al., 2020) and metabolic diseases (Ren and Anversa, 2015; van Niekerk et al., 2018; Wu N. N. et al., 2019; Almeida et al., 2020).

From the structural point of view, the neuron can be divided into three compartments: the soma, the axon and the dendrites. Axons can grow from a few micrometers up to many feet to reach their targets while dendrites are much shorter but can form highly branched nets. Since neurons are post-mitotic and long-lived cells, they must handle external stress removing aggregated and/or damaged proteins and organelles. For this reason, autophagy can perform this function in each neuronal compartment (Stavoe and Holzbaur, 2019). The interruption of homeostasis and physiological functions in the CNS, due to numerous pathophysiological events, can generate a wide range of diseases or pathological consequences. In particular, alteration of the redox state, inflammation, metabolic disorders, and failure in quality control of cellular proteins and organelles have been implicated in neurological and neurodegenerative disorders. In fact, a similar concept is also valid for psychiatric disorders (Menzies et al., 2015; Guo et al., 2018; Tomoda et al., 2020).

Numerous scientific studies have shown that the basal activity of autophagy is fundamental for the maintenance of homeostasis and neuronal vitality: in fact, it is known that neurons are particularly vulnerable in case of altered and/or switched off autophagy (Yamamoto and Yue, 2014; Kulkarni et al., 2018). It is important to stress that autophagy in CNS is important not only to maintain neuronal homeostasis, but also to ensure neurodevelopment (Stavoe and Holzbaur, 2019). Studies conducted in cultured embryonic peripheral neurons showed a retrogradely transport of large acidified vesicles from axons toward the soma along microtubules, suggesting that autophagosomes formed in the distal axon and were subsequently transported to the neuronal soma. The neuronal soma is rich in lysosomes and late endosomes with which autophagosomes fuse to lead autolysosomes (Maday and Holzbaur, 2014). Maintaining the integrity of proteins, neurotransmitters, receptors (localized at the synaptic level), and organelles (synaptic vesicles, mitochondria) is essential to support neuronal functionality (Lu et al., 2017; Conway et al., 2020).

Recent studies have revealed that autophagosomes are not only present in axons but also in the site of synaptic activation and these data suggest the possibility that neuronal autophagy regulates synaptic functions and neuroplasticity of the nervous system (Wang et al., 2015; Vanhauwaert et al., 2017; Tomoda et al., 2020). Synaptic plasticity requires intense biochemical activity that includes the synthesis of presynaptic neurotransmitters, postsynaptic receptor, signal transduction activity, gene expression, proper regulation of synapse, synaptic vesicle formation, and proper control of mitochondria (Binotti et al., 2015; Alvarez-Castelao and Schuman, 2015; Cohen and Ziv, 2017; Wang et al., 2017). All of these synaptic components are susceptible to wear and damage due to the frequencies of neuronal activation. Consequently, synapses are a site of great demand for cellular catabolic activities, for which an efficient degradation to support synaptic functions is indispensable (Vijayan and Verstreken, 2017). BDNF is also an inductor of long-term potentiation (LTP), considered one of the major cellular mechanisms that underlies learning and memory. Scientific evidence has shown that a receptor for BDNF is localized on the autophagosomic membrane, supporting the role of autophagy in transduction of BDNF signals (Nikoletopoulou et al., 2017; Nikoletopoulou and Tavernarakis, 2018). To date, it is known that failure of autophagy functions causes neuronal death, while impaired autophagy functions are associated with neurodegenerative disorders such as: Alzheimer’s disease, Huntington’s disease, and Parkinson’s disease (Menzies et al., 2017; Conway et al., 2020). Studies in mice with ablation of autophagy genes *ATG5* and *ATG7* showed degeneration of axonic terminals, progressive axonal swelling, and no signs of the autophagosome formation (Komatsu et al., 2006; Komatsu et al., 2007).

In addition, a recent study conducted on an *in vivo* model of knockout mice conditionally lacking the essential autophagy protein Atg5, showed that the loss of this neuronal mechanism led to a selective accumulation of the endoplasmic reticulum in axons. Increased endoplasmic reticulum leads to increased excitatory neurotransmission due to high release of calcium from the organelle and compromises neuronal viability. These results have suggested, therefore, that neuronal autophagy could control the axonal calcium reserves of endoplasmic reticulum to regulate neurotransmission in healthy neurons and in the brain (Kuijpers et al., 2021).

### Autophagy and Neurodegenerative Diseases

To date, it is known that failure of autophagy causes neuronal death, while impaired autophagy is associated with neurodegenerative disorders such as: Alzheimer’s Disease (AD), Huntington’s Disease (HD), Parkinson’s Disease (PD), and Amyotrophic Lateral Sclerosis (ALS) (Menzies et al., 2017; Conway et al., 2020). These pathologies are characterized by the formation of intracellular aggregates that result from misfolding and oligomerization of proteins. AD, the most common neurodegenerative disease, is characterized by extracellular β-amyloid plaques and intracellular neurofibrillary tangles composed of aggregated hyperphosphorylated tau protein (Hanzel et al., 2014; Cheignon et al., 2018). A dysfunctional autophagy is present in AD suffering patients, characterized by the loss of lysosome acidification, lysosomal alteration, failure to fusion between autophagosome and lysosomes and accumulation of autophagosomes (Yoon and Kim, 2016). Beclin1 is a key factor in the formation of autophagosomes and has been shown to be transcriptionally suppressed in the brain of AD (Salminen et al., 2013); in addition, caspase-3, an executive enzyme in the apoptosis pathway, can split the beclin1 protein and lead to the destruction of autophagy (Guo et al., 2018). Based on these observations, it is reasonable to assume that restoring lysosome function can increase the removal of protein aggregations. Finally, *in vitro* treatment with rapamycin, an enhancer of autophagy, has been shown to significantly increase the fusion of the autophagosomes with the lysosomes, improving the autophagic result (Li Q. et al., 2017).

PD, the second most common neurodegenerative disease, is characterized by selective loss of dopamine neurons in substantia nigra and accumulation of Lewy bodies, composed in misfolded and aggregated α-synuclein protein (Dauer and Przedborski, 2003; Lashuel et al., 2013). A close connection between this neurodegenerative disease and autophagy was indicated by the finding of dysfunctional lysosomes and accumulation of autophagosomes in the post-mortem brain samples of PD patients (Dehay et al., 2010; Xilouri et al., 2016). Additionally, if lysosomes are inhibited the levels of α-synuclein are increased, suggesting a link between α-synuclein degradation and autophagy (Guo et al., 2018). Lysosomal hydrolases, the enzymes responsible for degradation in lysosome, can be altered by autosomal recessive mutations: the consequence is the induction of defects in autophagosome-lysosome pathway and the aggregation of α-synuclein. Finally depletion of ATP6AP2, a transmembrane protein essential for lysosomal acidification, has been associated with PD (Abeliovich and Gitler, 2016).

HD is an autosomal dominant neurodegenerative disease, characterized by the repetition of trinucleotide CAG in the gene related to the protein huntingtin which leads to the expansion and pathogenic aggregation of the protein (Saudou and Humbert, 2016; Jimenez-sanchez et al., 2017). Huntingtin protein plays a key role in the autophagic process: in fact, its reduction results in an abnormal accumulation of autophagosomes (Wong and Holzbaur, 2014) and non-mutant huntingtin binds P62 to interact with ULK1 and activate it (Ravikumar et al., 2005).

ALS is a fatal disease characterized by the selective loss of motor neurons in the brain and spinal cord that causes weakness and muscle atrophy (Guo et al., 2018). The main cause of ALS is a mutation in *superoxide dismutase 1* and other genes that produce dysfunctional proteins, toxic cellular effects and oxidative stress (Medinas et al., 2018). To date, it is known that autophagy is closely associated with ALS (Morimoto et al., 2007); in fact, autophagic processes are also activated in degenerated motor neurons, although a reduced digestion of lysosomal load has been suggested (Sasaki, 2011). Finally, it has been highlighted that mutated and autophagy-related proteins are involved in the onset of ALS (Filimonenko et al., 2007). Scientific evidences indicate that dysregulated autophagy plays a key role in the neurodegenerative diseases; for this reason, the regulation of autophagy is a potential therapeutic strategy to improve the course of neurodegenerative diseases.

Autophagy is also involved in higher brain functions, such as learning, memory, mood, social interaction, and cognition. In fact, it has been pointed out that deregulated autophagy is responsible for the predisposition to neurological disorders such as schizophrenia, bipolar disorder, psychosis, attention deficit disorder, hyperactivity, autism, cognitive decline, and depression (Tomoda et al., 2020). In addition, autophagic activity is known to gradually decrease during aging, in conjunction with the reduction of these brain functions (Vilchez et al., 2014). This apparent correlation has been demonstrated by studies that have shown that the intake of substances that enhance autophagy, are responsible for an increase in life span. The same substances may reduce memory deterioration associated with aging (Eisenberg et al., 2009). Since autophagic regulation has been shown to alleviate the deficit in synaptic plasticity and improve cognition, its regulation can be therapeutic and diagnostic in neurological and neuropsychiatric disorders.

In summary, we can say that the strategies used by neuronal autophagy to ensure the proper functioning of the CNS are basically five (Tomoda et al., 2020):

• degradation of dysfunctional cytosolic proteins;
• degradation of damaged organelles;
• selective surface presentation of neurotransmitter receptors;
• degradation of neurotransmitter activity and their receptors;
• morphological and functional regulation of synapses.

### Autophagy and Metabolic Syndrome

Inflammatory diseases can trigger autophagy dysfunction (Deretic and Klionsky, 2018). It has been highlighted that autophagy is fundamental for the maintenance of cellular metabolic homeostasis and that it plays a crucial role in the control of body metabolism, whose dysregulation could participate in the onset of Mets (Lim et al., 2018). The role of autophagy in MetS was widely studied using animal models in which genetic alterations were induced (Lim et al., 2018). An example of this can be observed in a scientific study that highlighted how knockout mice of *ATG7*, an essential autophagy gene in pancreatic β-cells, showed structural and functional defects of these cells; with subsequent glucose intolerance and increased predisposition to develop diabetes (Ebato et al., 2008; Jung et al., 2008; Quan et al., 2012). Additionally, in another study, overexpression of *ATG5* another essential autophagic gene, improved the metabolic profile of older mice (Pyo et al., 2013). A further example of the effects of autophagy is confirmed by important results showing how systemic autophagic insufficiency is able to cause deterioration of adaptation to metabolic stress and facilitate progression from obesity to diabetes (Lim et al., 2014). Recently it has been shown that Mets is characterized by a dysfunctional mitophagy (Wu H. et al., 2019) and present alterations which manifest as inadequate acidification in lysosomes (Yamamoto et al., 2017; Park et al., 2020; Yamamoto et al., 2020).

## Conclusion

MetS is a clustering of several disorders including dyslipidemia, obesity and hyperglycemia/insulin resistance and every component is close connected with a high risk of developing atherosclerotic cardiovascular disease and type 2 diabetes. In addition, Mets has been shown to develop secondary disorders that affect the nervous system; the altered physiological state, due to Mets, can generate neurological deficits and a cognitive metabolic syndrome (Gasparova et al., 2018).

A physiopathological mechanism that links Mets and the neurological disorders is the endothelial dysfunction. In fact, in both cases, we can find an alteration of the endothelium of the cardiocirculatory system and of the BBB, respectively. In this review we wanted to better characterize the important correlation MetS-Neuronal disorders associated with another key process of cellular homeostasis: the autophagy. It has been highlighted that autophagy is fundamental for the maintenance of cellular metabolic homeostasis and that its dysregulation could participate in the onset of Mets (Lim et al., 2018). Moreover, neuronal autophagy is important also for the maintenance of homeostasis and vitality of neurons and these cells are particularly vulnerable in case of altered and/or switched off autophagy (Yamamoto and Yue, 2014; Kulkarni et al., 2018). It is important to stress that neuronal autophagy regulates also presynaptic excitatory neurotransmission by controlling the axonal cellular organelle ER. In particular, scientific experiments have shown that the deprivation of the Atg5, essential autophagic protein, leads to a selective accumulation of the tubular endoplasmic reticulum in axons, an increased release of the calcium ion, increased excitatory neurotransmission and impairment of postnatal viability *in vivo*. Therefore, the main consequence is an altered control of neuronal neurotransmission that, to occur properly, needs physiological autophagy (Kuijpers et al., 2021). Dysregulated autophagy can occur for multiple reasons but current scientific literature highlighted a common link in both Mets and neurological or neurodegenerative disorders. In fact, dysfunctional lysososomal acidification may be involved, leading to consequent failure of the fusion between autophagosomes and lysosomes (Yamamoto et al., 2017; Park et al., 2020; Yamamoto et al., 2020). Although this hypothesis is very fascinating, further studies are needed to confirm this link. In this direction, autophagy may be considered a target to reduce MetS risk factors and related disorders.

In summary, the model we propose is as follows:

• MetS involves, in addition to the risks already mentioned, the predisposition to the onset of neurological disorders;
• The inflammatory process is constantly present in MetS and participates in the onset of the neurological component. In fact, the subsequent dysfunction of the vascular endothelium involves the suffering of the nerve cells and the loss of integrity of the BBB;
• The autophagic process, notoriously involved in MetS, is a fundamental actor also in neurological disorders;
• Dysfunctional lysososomal acidification occurs in both MetS and neurological pathologies;
• Maintaining proper autophagic regulation, could reduce the constellation of primary and secondary MetS risk factors.

**FIGURE 3:**
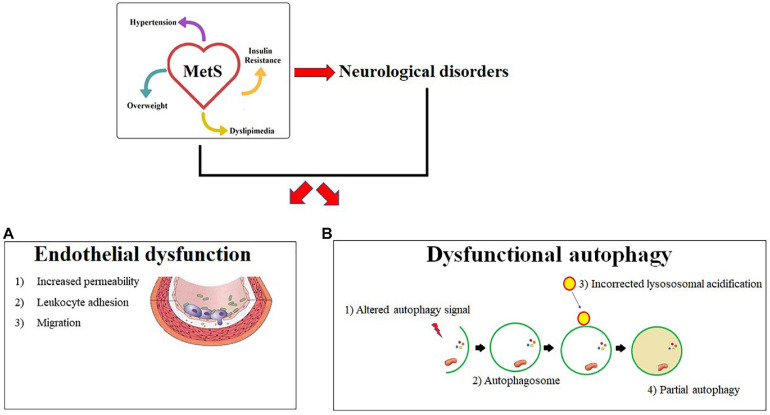
Common mechanisms in Mets and neurological diseases. MetS and its neurological complications are characterized by two common pathophysiological mechanisms described in panels a and b. In particular, **(A)** endothelial dysfunction is shown with the main characteristics: (1) Increased permeability (2) Leukocyte adhesion (3) Cellular Migration. **(B)** Shows a dysfunctional autophagy due to an altered signal. An incorrect lysosomal acidification is responsible for the changed formation of the autofagosoma-lisosome complex and a consequent partial autophagy.

A summary cartoon of this described model is shown in Figure 3.

## Author Contributions

JM and VMo conceptualized, designed, and wrote the manuscript. MG, CC, VMu, SN, FB, FS, RM, SR, MZ, and CM revised the manuscript critically. All authors contributed to the article and approved the submitted version.

## Conflict of Interest

The authors declare that the research was conducted in the absence of any commercial or financial relationships that could be construed as a potential conflict of interest.
