# Supplementary figures and images for: From Metabolic Syndrome to Neurological Diseases: Role of Autophagy
Source: Front Cell Dev Biol. 2021 Mar 19;9:651021. doi: 10.3389/fcell.2021.651021 (PMC8017166; doi:10.3389/fcell.2021.651021)

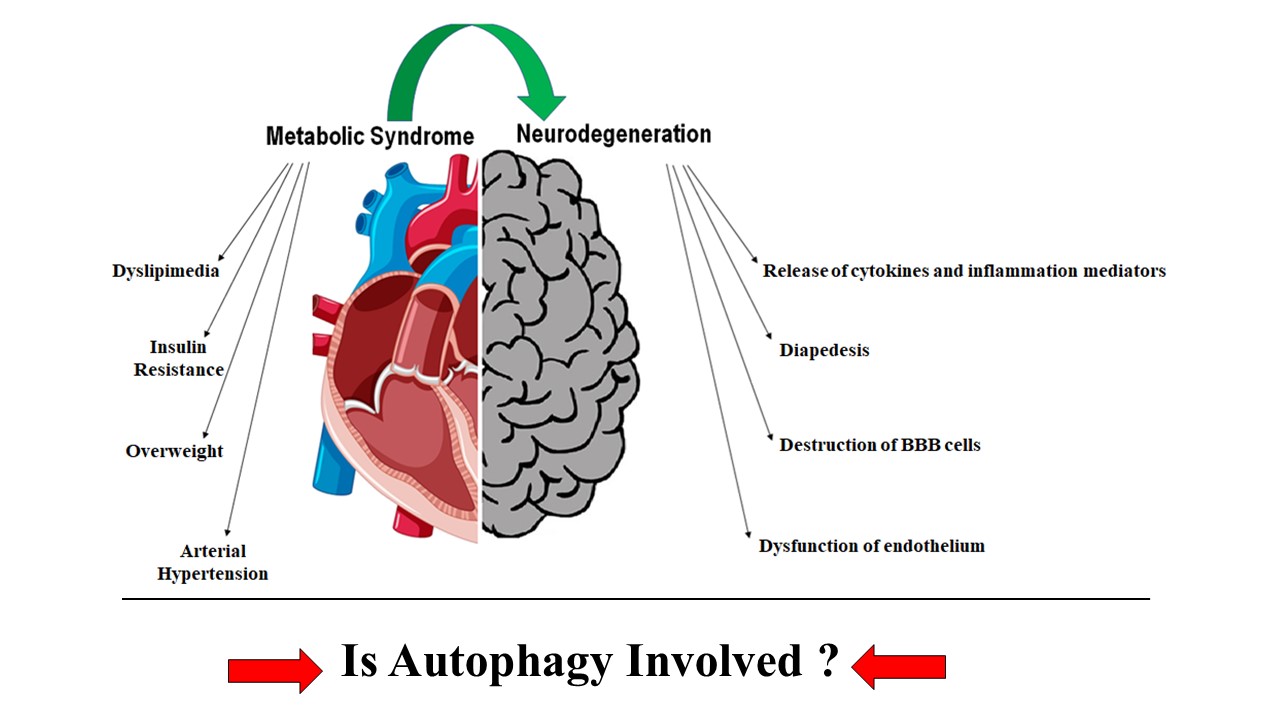

Supplement: Supplementary file 1 [file Image_1.JPEG]
